# Heterocyclization of polarized system: synthesis, antioxidant and anti-inflammatory 4-(pyridin-3-yl)-6-(thiophen-2-yl) pyrimidine-2-thiol derivatives

**DOI:** 10.1186/s13065-018-0437-y

**Published:** 2018-06-08

**Authors:** Wesam S. Shehab, Magda H. Abdellattif, Samar M. Mouneir

**Affiliations:** 1 Department of Chemistry, Faculty of Science, Zagazig University, Zagazig, 44519 Egypt; 2 Department of Pharmaceutical Chemistry, Deanship of Scientific Research, Taif University, Taif, 21974-888 Kingdom of Saudi Arabia; 3 Departments of Pharmacology, Faculty of Veterinary Medicine, Cairo University, Cairo, 12211 Egypt

**Keywords:** Pyrazolopyrimidine, Thiophene, Chalcone, Pyrazol, Pyranone, Anti-inflammatory-antioxidant-cycloxygenase-5-LOX-DPPH

## Abstract

**Background:**

Chalcones are intent in the daily diet as a favorable chemotherapeutic compound; on the other hand thiophene moiety is present in a large number of bioactive molecules having diverse biological efficiency.

**Results:**

Our current goal is the synthesis of (*E*)-1-(pyridin-3-yl)-3-(thiophen-2-yl) prop-2-en-1-one **3** that^’^s used as a starting compound to synthesize the novel pyrimidine-2-thiol, pyrazole, pyran derivatives. Chalcones **3** was prepared by condensation of 3-acetylpyridine with thiophene 2-carboxaldehyde which reacted with thiourea to obtain pyrimidinthiol derivative **4**. Compound **4** was allowed to react with hydrazine hydrate to afford 2-hydrazinylpyrimidine derivative **5**. Compound **5** was used as a key intermediate for a facile synthesis of the targets **6** and **7**. In contrast, pyranone **8** was obtained by transformation of compound **5**. Using as a precursor for the synthesis of new pyrazolo pyrimidine derivatives **9**–**10**. The major incentive behind the preparation of these compounds was the immense biological activities associated to these heterocyclic derivatives.

**Conclusions:**

The newly synthesized compounds (**1**–**4**) showed potent anti-inflammatory activities both in vitro and in vivo. They also exhibited promising antioxidant vitalities against α, α-diphenyl-β-picrylhydrazyl scavenging activity and lipid peroxidation. In conclusion, compound **1** showed a hopefully anti-inflammatory and antioxidant activities.
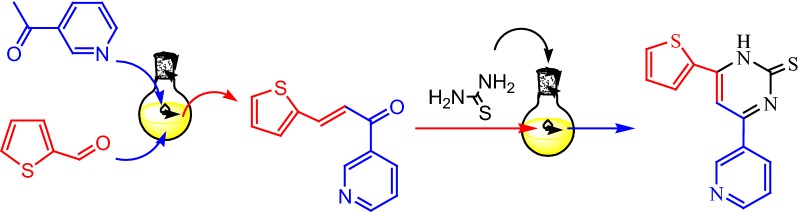

## Introduction

Chalcones are distinguished by their easy synthesis from Claisen-Schmidt condensation. The chemical structure of chalcones formed of two aromatic rings joined by a thee carbon, α, *β*-unsaturated carbonyl system (1, 3-diphenylprop-2-en-1-one) [1, 2]. They have been authenticated with diverse biological efficiency including antibacterial [3–8], anti-inflammatory [9–12], antioxidant [13–16], anti-tumor effects [17–22]. Also, pyridine derivatives of different heterocyclic nucleus have shown potent pharmacological properties like cytotoxic activity [23, 24]. Recent studies have demonstrated that chalcones are target in the daily diet as a favorable chemotherapeutic compounds [25] and anti-proliferative activity [26]. On the other hand thiophene moiety is present in a large number of bioactive molecules having diverse biological activities such as anti-inflammatory [27], anticonvulsant [26], antimicrobial [27] and antitumor [28]. Moreover, thiophene moiety is a well-known isostere for benzene; for example, the replacement of benzene ring of the antidepressant drug, Viloxazine led to a prolongation of the half-life [29]. Recently we were concerned with the synthesis of polyfunctional heterocyclic compounds, where the (*E*)-1-(pyridin-3-yl)-3-(thiophen-2-yl) prop-2-en-1-one **3** was used as a starting compound. The remarkable biological activity of the polycyclic heterocyclic compounds encouraged us to continue our previous work on the synthesis of fused pyrimidine [30–33] and their applications, by designing a polycyclic heterocyclic compounds containing five and/or six rings fused with each other to develop a superior biological activity.

## Results and discussion

### Chemistry

**Scheme 1:**
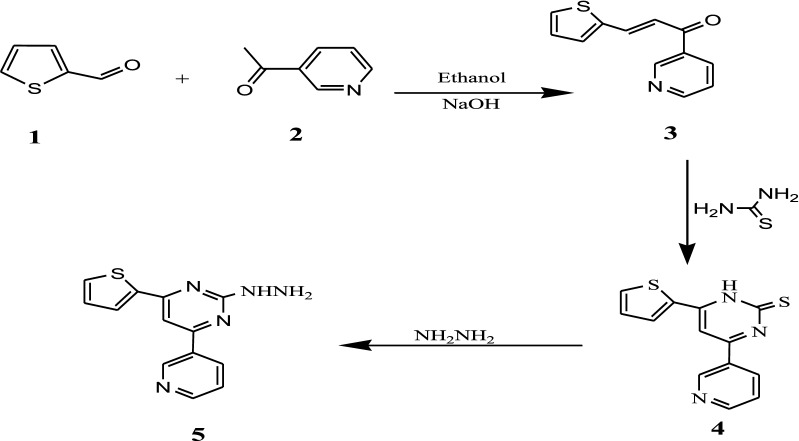
Synthesis of pyrimidine derivatives

Aldol condensation reaction of 3-thiophenecarboxaldehyde **1** with 3-acetylpyridine **2** in ethanolic NaOH solution afforded chalcone **3**. The structure of compound **3** was elucidated by its IR, ^1^H NMR and ^13^C NMR. Its IR spectrum showed a characteristic peak for a conjugated carbonyl group at 1633 cm^−1^, and by its ^1^H NMR which gave signals at δ 7.53 (d, 1H, *J *= 12.9 Hz, (CH=C–C=O), and 7.92 (d, 1H, *J *= 12.9 Hz (CH=C–C=O) and two doublet signals at δ = 7.28 and 7.94 due to thiophenyl-*C*_*4′*_*H* and thiophenyl-*C*_*3′*_*H* and another at 8.11 owing to thiophenyl-*C*_*5′*_*H* whereas, the ^13^C NMR spectrum showed a signal at (δ in ppm) 123 caused by ethylene group and 125, 126, 135, 149 and a signal due to C=O groups at 193. [3 + 3] base induced cycloaddition of chalcone **3** with thiourea gave 4-(pyridin-3-yl)-6-(thiophen-2-yl) pyrimidine-2(1H)-thione **4**. IR spectra of compounds **4** showed the presence of a C=S band at 1270 cm^−1^ and an absorption band in the range 3433–3490 cm^−1^ attributed to the amine (NH). The ^1^H NMR spectrum of compound **4** two doublet signals at δ = 7.28 and 7.94 due to thiophenyl-*C*_*4′*_*H* and thiophenyl-*C*_*3′*_*H* and another at 8.11 as a result of thiophenyl-*C*_*5′*_*H*. The spectra displayed a singlet at 8.82 for NH, respectively. The hydrazinopyrimidine derivative **5** was synthesized by condensation of the thiopyrimidinone **4** with hydrazine hydrate in refluxing alcohol, the structure of compound **5** was confirmed by the IR, ^1^H NMR and elemental analysis, where its IR revealed the absorption bands at ν max = 3212 for the NH_2_ and 3184 cm^−1^ for the NH group, ^1^H NMR spectrum gave the signals at δ = 8.93–8.95 as a broad singlet for NH_2_, hydrazine NH, respectively (Scheme 1).

**Scheme 2:**
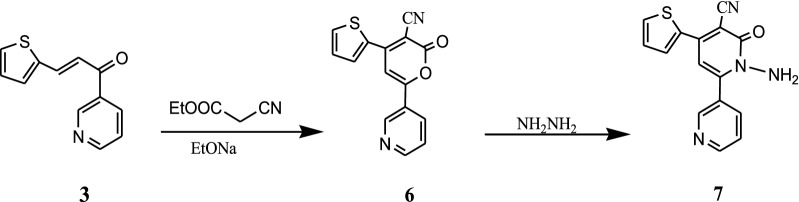
Synthesis of pyranone and pyridinones derivatives

Cyclocondensation of chalcone **3** and ethyl cyanoacetate in the presence of sodium ethoxide under the reflux conditions [33] gave pyranone derivative **6**. Condensation with hydrazinehydrate [34, 35] in refluxing ethanol leads to ring transformation producing corresponding pyridinones **7**. The structure of the target **7** was confirmed from its spectral data, where is IR spectra showed absorption bands in the region 2222 and 1688 cm^−1^ characteristic for C≡N and carbonyl group, respectively (Scheme 2).

**Scheme 3:**
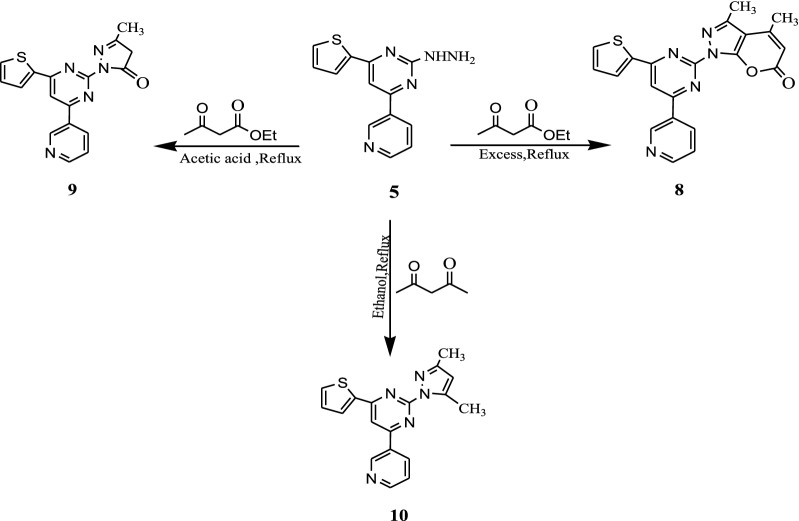
Synthesis of isolated and fused pyrimidine derivatives

The hydrazinopyrimidine derivative **5** was used as a precursor for the synthesis of some heterocyclic compounds. The hydrazinopyrimidine derivative **5** reacted with ethyl acetoacetate in excess manner to afford compound **8**. The formation of **8** may be proceeds via the formation of pyrazolone derivative **9** followed by the attack of methylene anion of pyrazolone to ketonic function of ethyl acetoacetate followed by pyran cyclization. IR spectrum of compound **8** revealed the absorption peaks at 1715 cm^−1^ characteristic of C=O groups respectively, ^1^H NMR exhibited the two singlets at δ = 2.25 and 2.32 for 2 CH_3_ protons and a singlet at δ = 5.60 ppm for pyranone H. Furthermore, the pyrimidine pyrazolone compound **9** was obtained as a result of attack of hydrazinofunction of **5** to ethyl acetoacetate. The pyrazolo pyrimidine **10** was synthesized by heating an alcoholic solution of compound **5** (10.0 mmol.) with acetylacetone (10.0 mmol.) at reflux temperature for 5 h. The IR spectra of **9** and **10** showed the disappearance of the hydrazine group where the ^1^H NMR spectrum showed singlet pyrimidine H at δ = 8.95 ppm and two singlets for the two CH_3_ protons, respectively (Scheme 3).

### Biological activity studies

#### In vitro anti-inflammatory activity

##### In vitro COX-1 and COX-2 inhibition

Compounds (**3**–**6**) were calorimetrically evaluated for their anti-inflammatory activities in vitro for COX-1 and COX-2 at 590 nm using ovine COX-1/COX-2 inhibitor screening assay kit [36]. Celecoxib was used as a standard reference drug.

##### In vitro 5-LOX inhibition

A bnova 5 lipoxygenase inhibitor screening assay was used [37]. Meclofenamate sodium was used as a standard reference drug. Results were expressed in Table 1 as IC _50_ as means of thee determinations the selectivity index was calculated also as IC_50_ (COX-1)/IC_50_ (COX-2).

**Table 1:** Display the anti-inflammatory activity of the newly synthesized compounds as IC _50_, µM for COX-1, COX-2 and 5-LOX

Group	IC _50_ (µM) COX-1	IC _50_ (µM) COX-2	COX-1/COX-2	IC 50 (µM) 5 LOX
Celecoxib	5.47	0.86	7.91	N.D
Meclofenamate sodium	ND	ND	ND	6.15
Compound **3**	3.7	0.39	9.49	4.71
Compound **4**	4.02	0.44	9.13	4.91
Compound **5**	4.60	0.87	5.29	6.98
Compound **6**	4.95	0.84	5.89	7.65

#### In vivo anti- inflammatory activity

Carrageenan induced rat paw edema in rats: Fifty rats were divided into ten groups (i.e., each group, five rats). The first group (control), received carboxymethyl cellulose. The second group was given diclofenac sodium as a standard anti-inflammatory drug. Groups (3–10) were orally given the newly synthesized compounds (**3**–**6**) in two dosages (5 and 10 mg/kg). Results were expressed as rat paw edema percent. One hour later after administration of tested doses, carrageenan was injected sub planter in the left hind footpad of each rat as 0.05 ml of 1% solution in sterile distilled water. Plethysmometer was used to measure paw edema volume from 0 to 4 h after carrageenan injection. Paw edema volume was compared with vehicle control group and reduction percent was calculated as the following

$$\% {\text{ reduction in edema}} = \left( {1 - {\text{ Vt Vc}}} \right) \, \times \, 100$$

Where Vt and Vc are the edema volume in the group treated with drug and control, respectively [38]. Results were expressed as mean ± standard deviation (SD). Differences between means were tested for significance using a one-way analysis of variance (ANOVA) followed by Duncan’s test (Table 2).

**Table 2:** Inhibition percent of rat paw edema after administration of newly synthesized compounds

Groups	0 h	1 h	2 h	3 h	4 h
Diclofenac sodium	0.49 ± 0.032^a^	30.22 ± 1.27^a^	33.85 ± 1.19^a^	36.21 ± 0.93^a^	41.10 ± 3.98^a^
Compound **3** 5 mg/kg.b.wt	0.48 ± 0.027^ab^	21.72 ± 0.79^b^	22.79 ± 1.07^d^	24.79 ± 0.49^c^	28.41 ± 1.30^c^
Compound **3** 10 mg/kg.b.wt	0.47 ± 0.04^ab^	31.98 ± 9.35^a^	29.93 ± 1.43^b^	34.16 ± 0.61^b^	41.15 ± 0.750^a^
Compound **4** 5 mg/kg.b.wt	0.46 ± 0.03^b^	18.91 ± 1.19^bc^	20.07 ± 1.43^e^	21.96 ± 1.25^d^	27.38 ± 1.68^c^
Compound **4** 10 mg/kg.b.wt	0.49 ± 0.01^ab^	21.43 ± 0.96^b^	26.27 ± 49^e^	33.13 ± 2.64^b^	37.15 ± 0.69^b^
Compound **5** 5 mg/kg.b.wt	0.49 ± 0.01^a^	11.62 ± 1.24^de^	14.61 ± 1.81^g^	18.41 ± 1^e^	19.48 ± 0.89^e^
Compound **5** 10 mg/kg.b.wt	0.48 ± 0.01^ab^	15.41 ± 0.83^cd^	18.83 ± 0.75^ef^	24.19 ± 1.59^c^	28.59 ± 2.26^c^
Compound **6** 5 mg/kg.b.wt	0.50 ± 0.02^a^	9.61 ± 1.12^e^	12.28 ± 1.38^h^	17.19 ± 1.26^e^	18.51 ± 2.26^e^
Compound **6** 10 mg/kg.b.wt	0.49 ± 0.01^a^	13.75 ± 1.15^de^	17.52 ± 1.13^f^	20.43 ± 0.65^d^	22.39 ± 1.16^d^

Values are expressed as mean ± SD

Different superscript letters are significantly different at P ≤ 0.05

#### Antioxidant screening

- DPPH free radical scavenging assay was determined (4). Results were presented in Table 3 as IC _50_ (μg/ml). Ascorbic acid was used as reference standard antioxidant.

**Table 3:** Showing antioxidant activities of the newly synthesized compounds

Groups	IC_50_ (μg/ml) for DPPH Scavenging	IC_50_ (μg/ml) for anti-lipid peroxidation
Compound **3**	10.72 ± 0.54	16.81 ± 2.71
Compound **4**	12.64 ± 0.41	22.53 ± 3.25
Compound **5**	14.61 ± 0.72	23.62 ± 2.31
Compound **6**	15.26 ± 0.44	22.67 ± 3.51
Ascorbic acid	13.71 ± 0.75	25.72 ± 1.23
- Lipid peroxidation assay (**5** and **6**) was calculated as IC_50_ and recorded in Table 3.

The newly synthesized compounds exhibited a remarkable in vivo and in vitro anti-inflammatory activity. These results are in agreement with those obtained by other researchers [39]. They reported that some novel pyrimidine-pyridine hybrids inhibited cyclooxygenase enzyme and had a significant anti-inflammatory activity comparable to celecoxib as a standard drug. In this concern, other authors [40] reported an investigation of the efficacy of pyridine and pyrimidine analog of acetaminophen as peroxyl radical trapping antioxidants and inhibitors of enzyme catalyzed lipid peroxidation by cyclooxygenase and lipoxygenase. Compounds **3** and **4** exhibited antioxidant activity screening higher scavenging activity towards the DPPH radicals than that of ascorbic acid. Similar results were reported for new pyridine and triazolopyridine derivatives [41–45].

## Experimental

### Chemistry

Melting points were measured using an Electrothermal IA 9100 equipment with open capillary tube and were kept uncorrected. All experiments were done using dry solvents. TLC was performed on Merck Silica Gel 60F254 with detection by way of UV Light. The formed compound has been purified using recrystallization. The IR spectra (KBr disc) were recorded using Pye Unicam Sp-3-300 or a Shimadzu FTIR 8101 PC infrared spectrophotometer. The ^1^H NMR and ^13^C NMR spectra were measured by means of JEOL-JNM-LA 400 MHz spectrometer using DMSO-d_6_ as a solvent. All chemical shifts had been expressed on the δ (ppm) scale using TMS as an internal well-known reference. The coupling constant (*J*) values are given in Hz. Analytical information was acquired from the Microanalysis center at Cairo University, Giza, Egypt.

#### (*E*)-1-(pyridin-3-yl)-3-(thiophen-2-yl) prop-2-en-1-one (3)

To a stirred mixture of thiophene-2-carbaldehyde **1** (100 mmol) and 3-acetylpyridine **2** (100 mmol) in 200 ml ethanol at room temperature, 40% NaOH aqueous solution was added portion-wise while stirring 2 h. The pale yellow precipitate formed was filtered and washed using 4% aqueous HCl, and crystallized from ethanol to give chalcone **3** in 82% yield, mp 256–258 °C. IR (KBr) cm^−1^: 3336, 3255, 1678, 1645; ^1^H NMR (300 MHz, DMSO-d6): 6.93–6.96 (t, 1H, H5′-pyridine), 7.47–7.51 (dd, 1*H*, *H*3′-pyridine), 7.30–7.327(t, 1H, H4′-pyridine), 7.53 (d, 1H, *J *= 12.9 Hz, (C=O)(CH=C), 7.92 (d, 1H, *J *= 12.9 Hz (C=O) (C=CH), δ = 7.28(d, 1H, *J* = 3.6 Hz, thienyl-*C*_*3*_*′H*), 7.94 (dd, 1H, thienyl-*C*_*4*_*′H*), 8.11 (d, 1H, *J* = 5.2 Hz, thienyl-*C*_*5*_*′H*).^13^C NMR (DMSO-*d*_6_, 150 MHz): δ = 200.18 (C1=O);153.3 (C4′-pyridine); 149.2 (C2′-pyridine); 147.4 (C3); 135.2 (C4′′); 134.9 (C6′-pyridine); 133.2 (C′′-pyridine); 132.8 (C2′′); 127.1(C2); 126.8 (C5′-pyridine);125.4 (C3′′);123.6(C1′′). Anal. Calcd for C_12_H_9_NOS (215.27): C, 66.95; H, 4.21; N, 6.51; S, 14.90; Found C, 66.89; H, 4.19; N, 6.50; S, 14.79%.

#### 4-(pyridin-3-yl)-6-(thiophen-2-yl) pyrimidine-2(1*H*)-thione (4)

Chalcone **3** (10 mmol) was added to sodium ethoxide solution [prepared from sodium metal (0.23 g, 10 mmol) and 50 ml of absolute ethanol] then thiourea (10 mmol) was added. The reaction mixture was refluxed for 16 h., left to cool and poured into crushed ice and neutralized with diluted hydrochloric acid, filtration, washed with ethanol and dried. Crystallization from EtOH afforded the pyrimidine derivatives **4**. Yellow powder, yield 74%, mp 220–225 °C; IR (KBr): 3433 (NH), 1270 (C=S) cm^−1^; ^1^H NMR (300 MHz, DMSO-d_6_): δ = 6.93–6.96 (t, 1H, H5′-pyridine), 7.47–7.51 (dd, 1*H*, *H*3′-pyridine), 7.30–7.327(t, 1H, H4′-pyridine), 7.28(d, 1H, *J* = 3.6 Hz, thienyl-*C*_*3*_*′H*), 7.94 (dd, 1H, thienyl-*C*_*4*_*′H*), 8.11 (d, 1H, *J* = 5.2 Hz, thienyl-*C*_*5*_*′H*), 8.82 (s, D_2_O-exchangeable, 1H, pyrimidin NH). ^13^CNMR (DMSO-d_6_, 100 MHz) δ: 110.2, 123.9, 127.1, 128.2, 130.5, 136.6, 137.2, 151.5, 152.0, 157.164.6, 180.4. Anal. Calcd for C_13_H_9_N_3_S_2_ (271.36): C, 57.54; H, 3.34; N, 15.48; S, 23.63; Found: C, 57.49; H, 3.32; N, 15.49; S, 23.59%.

#### 1-(4-(pyridin-3-yl)-6-(thiophen-2-yl)pyrimidin-2-yl)hydrazine (5)

The reaction of thiopyrimidinone **4** (10.0 mmol) with hydrazine hydrate (10.0 mmol) catalyzed by acetic acid (5 drops) in refluxing ethanol for 6 h. Evaporation of alcohol and recrystallization with ethanol gave compound **5** as pale brown crystals mp 180–182 °C, yield 85%. IR: νmax/cm^−1^: 3212 (NH_2_), 3184 (NH). ^1^H NMR (DMSO-d_6_): δ = 6.93–6.96 (t, 1H, H5′-pyridine), 7.47–7.51 (dd, 1*H*, *H*3′-pyridine), 7.30–7.327(t, 1H, H4′-pyridine), δ = 7.28(d, 1H, *J* = 3.6 Hz, thienyl-*C*_*3*_*′H*), 7.53 (dd, 1H, thienyl-*C*_*4*_*′H*), 7.89 (d, 1H, *J* = 5.2 Hz, thienyl-*C*_*5*_*′H*) 0.7.94 (s, 1H, pyrimidin), 8.93–8.95 (brs, 3H, D_2_O Exch., NH_2_, NH). ^13^C NMR (DMSO-d_6_, 100 MHz) δ: 98.4, 123.9, 127.1, 128.2, 130.5, 134.1, 137.2, 151.5, 152.0, 148.0, 149.1, 155.8, 157, 161.1. Anal.Calcd. For C_13_H_11_N_5_S (269.32): C, 57.97; H, 4.12; N, 26.00; S, 11.91; Found: C, 57.96; H, 4.09; N, 26.03%.

#### 2-oxo-6-(pyridin-3-yl)-4-(thiophen-2-yl)-2*H*-pyran-3-carbonitrile (6)

To a stirred solution of chalcone **3** (10 mmol) and ethyl cyanoacetate (10 mmol) in 50 ml absolute ethanol, a sodium ethoxide solution prepared from 0.23 g sodium metal (10 mmol) and 10 ml absolute ethanol was added refluxing the reaction mixture for 8 h. The solid that formed after cooling was collected by filtration, washed with water, dried and finally crystallized from ethanol to afford compound **6** as pale yellow crystals in 72% yield, mp 210–212 °C; IR (KBr): 2222 (C≡N), 1688 (C=O) cm^−1^; ^1^H NMR (300 MHz, DMSO-d6): δ = 7.32 (s, 1H, C5), 6.93–6.96 (t, 1H, H5′-pyridine), 7.47–7.51 (dd, 1*H*, *H*3′-pyridine), 7.30–7.327(t, 1H, H4′-pyridine), 7.28(d, 1H, *J* = 3.6 Hz, thienyl-*C*_*3*_*′H*), 7.94 (dd, 1H, thienyl-*C*_*4*_*′H*), 8.11 (d, 1H, *J* = 5.2 Hz, thienyl-*C*_*5*_*′H*). ^13^C-NMR (DMSO-d_6_, 100 MHz) δ: 98.8, 113.3, 115.9, 123.8, 127.1, 128.2, 130.5, 137.7, 136.6, 149.6, 157.4, 173.4. Anal. Calcd for C_15_H_8_N_2_O_2_S (280.3): C, 64.27; H, 2.88; N, 9.99; S, 11.44; Found: C, 64.25; H, 2.84; N, 9.96; S, 11.42%.

#### 1-amino-6-oxo-4-(thiophen-2-yl)-1,6-dihydro-[2,3′-bipyridine]-5-cabonitrile (7)

To a solution of the pyranone **6** (2 mmol) in 30 ml of ethanol, hydrazine hydrate (2 mmol) was added. The mixture was refluxed for 6 h. Left to cool, the formed solid product was filtered off, dried, and then crystallized from ethanol to give compounds **7**. Yellow powder, yield 70%, mp 250–252 °C; IR (KBr): 3320, 3190 (NH_2_, NH), 2219 (C≡N), 1670 (C=O) cm^−1^; ^1^H NMR (300 MHz, DMSO-d_6_): δ = 2.51(s, D_2_O-exchangeable, 2H, NH_2_), 6.93–6.96 (t, 1H, H5′-pyridine), 7.47–7.51 (dd, 1*H*, *H*3′-pyridine), 7.30–7.327(t, 1H, H4′-pyridine), 7.28(d, 1H, *J* = 3.6 Hz, thienyl-*C*_*3*_*′H*), 7.94 (dd, 1H, thienyl-*C*_*4*_*′H*), 8.11 (d, 1H, *J* = 5.2 Hz, thienyl-*C*_*5*_*′H*). ^13^C-NMR (DMSO-d_6_, 100 MHz) δ: 110.8, 115.9, 121.3, 123.8, 127.1, 128.2, 130.5, 131.6, 136.8, 149.6, 150.0, 160.5, 169.4. Anal. Calcd for C_15_H_10_N_4_OS (294.33): C, 61.21; H, 3.42; N, 19.04; S, 10.89; Found: C, 61.20; H, 3.40; N, 19.09; S, 10.90%.

#### 3,4-dimethyl-1-(4-(pyridin-3-yl)-6-(thiophen-2-yl)pyrimidin-2-yl)pyrano[2,3-c]pyrazol-6(1*H*)-one (8)

Compound **5** (10 mmol) and ethyl acetoacetate in excess (30 ml) was heated at reflux temperature for 6 h. The mixture was poured into ice cold water and the obtained product washed with water, dried and recrystallized from ethanol to give pale brown crystals of the pyrazolone compound **8** mp 190–192 °C, yield 80% IR: νmax/cm^−1^ 3367 (NH), 1715 (C=O). ^1^H NMR (DMSO-d_6_): δ 2.25 (s, 3H, CH_3_), 2.32 (s, 3H, CH_3_), 5.60 (s, 1H, pyranone), 6.93–6.96 (t, 1H, H5′-pyridine), 7.47–7.51 (dd, 1*H*, *H*3′-pyridine), 7.30–7.327(t, 1H, H4′-pyridine), 7.28(d, 1H, *J* = 3.6 Hz, thienyl-*C*_*3*_*′H*), 7.94 (dd, 1H, thienyl-*C*_*4*_*′H*), 8.11 (d, 1H, *J* = 5.2 Hz, thienyl-*C*_*5*_*′H*). ^13^C-NMR (DMSO-d_6_, 100 MHz) δ: 11.9, 21.2, 101.5, 105, 118.5, 124.0, 125.5, 127.9, 33.1, 134.1, 141.7, 148.0, 149.1, 152.8, 155.6, 159.0, 160.9, 161.0. Anal. Calcd. For C_21_H_15_N_5_O_2_S (401.44): C, 62.83; H, 3.77; N, 17.45; S, 7.99; Found: C, 62.79; H, 3.79; N, 17.47; S, 7.95%.

#### 3-methyl-1-(4-(pyridin-3-yl)-6-(thiophen-2-yl) pyrimidin-2-yl)-1*H*-pyrazol-5(4*H*)-one (9)

Compound **5** (10 mmol) and ethyl acetoacetate (10 mmol) in acetic acid (30 ml) was heated at reflux temperature for 6 h. The mixture was poured into ice cold water and the obtained product washed with ice cold water, dried and recrystallized from ethanol to afford pale brown crystals of **9** mp 225–227 °C, yield 76% IR: νmax/cm^−1^: 3216 (NH), 1718 (C=O). ^1^H NMR (DMSO-d6): δ 1.20 (s, 3H, CH_3_), 2.25 (s, 2H, CH_2_, pyrazol), 6.93–6.96 (t, 1H, H5′-pyridine), 7.47–7.51 (dd, 1*H*, *H*3′-pyridine), 7.30–7.327(t, 1H, H4′-pyridine), 7.28(d, 1H, *J* = 3.6 Hz, thienyl-*C*_*3*_*′H*), 7.94 (dd, 1H, thienyl-*C*_*4*_*′H*), 8.11 (d, 1H, *J* = 5.2 Hz, thienyl-*C*_*5*_*′H*), 8.90 (s, 1H, pyrimidin).^13^C-NMR (DMSO-d_6_, 100 MHz) δ: 24.6, 42.4, 99.9, 124.0, 125.5, 127.6, 133.1, 134.1, 140, 148.0, 149.1, 156.1, 160.2, 163.1, 159.5, 172.8. Anal. Calcd. For C_17_H_13_N_5_OS (335.38): C, 60.88; H, 3.91; N, 20.88; S, 9.56; Found: C, 60.90; H, 3.90; N, 20.86; S, 9.55%.

#### 2-(3,5-dimethyl-1*H*-pyrazol-1-yl)-4-(pyridin-3-yl)-6-(thiophen-2-yl)pyrimidine (10)

A solution of compound **5** (10 mmol) in absolute ethanol and acetylacetone (10 mmol) was heated at reflux temperature for 5 h. the obtained product was recrystallized from ethanol to afford pale brown crystals of pyrazolo pyrimidine derivative **10**. mp 190–188 °C, yield 70% IR: νmax/cm^−1^ 3170 (NH), 1600 (C=N), 1574 (C=N). ^1^H NMR (DMSO-d6): δ 2.3 (s, 3H, CH_3_), 2.58 (s, 3H, CH_3_), 6.99 (s, 1*H*, pyrazole), 6.93–6.96 (t, 1H, H5′-pyridine), 7.47–7.51 (dd, 1*H*, *H*3′-pyridine), 7.30–7.327(t, 1H, H4′-pyridine), 7.28(d, 1H, *J* = 3.6 Hz, thienyl-*C*_*3*_*′H*), 7.94 (dd, 1H, thienyl-*C*_*4*_*′H*), 8.11 (d, 1H, *J* = 5.2 Hz, thienyl-*C*_*5*_*′H*), 8.94 (s, 1H, pyrimidine).^13^C-NMR (DMSO-d6, 100 MHz) δ: 11.1, 18, 105, 125.5, 127.6, 133.1, 140, 144.3, 148.0, 149.1, 155.6, 159.0, 161.0. Anal. Calcd. For C_18_H_15_N_5_S (333.41): C, 64.84; H, 4.53; N, 21.01; S, 9.62; Found: C, 64.80; H, 4.52; N, 21.02, S, 9.60%.

## Conclusions

We have reported the synthesis of (E)-1-(pyridin-3-yl)-3-(thiophen-2-yl) prop-2-en-1-one **3** and using to designing a polycyclic heterocyclic compounds containing five and/or six rings fused. Moreover, we concluded that compounds **3** and **4** showed a significant antioxidant activity regarding cyclooxygenase inhibitory activity, compound 3 presented the highest inhibitory activity in comparison to the standard reference drug [IC_50_ as 3.7 and 0.39 µM for COX-1 and COX-2, respectively compared to 5.47 and 0.86 for the standard celecoxib]. Compound **4** also showed a potent inhibitory activity for COX-2 with IC_50_ 0.44. Compounds **5** and **6** showed inhibitory activity against COX-1 and COX-2 nearly like that of the standard drug. Compound 3 showed the highest inhibitory potential for 5-lipoxygenase with IC_50_ (4.71 µM) compared to (6.15 µM) of the standard anti-inflammatory drug meclofenamate sodium.

## Abbreviations

EtOH: ethanol
NMR: nuclear magnetic resonance
IR: infrared radiation
DMSO: dimethyl sulfoxide
COX-1: cyclooxygenase-I
COX-2: cyclooxygenase-II
5-LOX: 5-lipoxygenase
DPPH: 2, 2-diphenyl-1-picrylhydrazil

## References

[1] Firoozpour L, Edraki N, Nakhjiri M, Emami S, Safavi M, Ardestani SK, Khoshneviszadeh M, Shfiee A, Foroumadi A (2012). Cytotoxic activity evaluation and QSAR study of chromene—based chalcone. Arch Pharm Res.

[2] Baviskar BA, Baviskar B, Shiradkar MR, Deokate UA, Khadabadi SS (2009). Synthesis and antimicrobial activity of some novel. Benzimidazolyl chalcones. Eur J Chem.

[3] Munawar MA, Azad M, Siddiqui HL (2008). Synthesis and antimicrobial studies of some quinolinylpyrimidine derivatives. J Chin Soc.

[4] Azad M, Munawar MA, Siddiqui HL (2007). Antimicrobial activity and synthesis of quinoline-base chalcones. J Appl Sci.

[5] Kalirajan R, Sivakumar SU, Jubie S, Gowramma B, Suresh B (2009). Synthesis and biological evaluation of some heterocyclic derivatives of chalcones. Int J ChemTech Res.

[6] Talia JM, Debattista NB, Pappano NB (2011). New antimicrobial combinations: substituted chalcones-oxacillin against methicillin resistant *Staphylococcus aureus*. Braz J Microbiol.

[7] Eumkeb G, Siriwong S, Phitaktim S, Rojtinnakorn N, Sakdarat S (2012). Synergistic activity and mode of action of flavonoids isolated from smaller galangal and amoxicillin combinations against amoxicillin-resistant *Escherichia coli*. J Appl Microbiol.

[8] Do TH, Nguyen DM, Truong VD, Do THT, Le MT, Pham TQ, Thai KM, Tran TD (2016). Synthesis and selective cytotoxic activities on rhabdomyosarcoma and noncancerous cells of some heterocyclic chalcones. Molecules.

[9] Sato Y, Shibata H, Arakaki N, Higuti T (2004). 6,7-dihydroxyflavone dramatically intensifies the susceptibility of methicillin-resistant or -sensitive *Staphylococcus aureus* to beta-lactams. Antimicrob Agents Chemother.

[10] Babasaheb PB, Sachin AP, Rajesh NG (2010). Synthesis and biological evaluation of nitrogencontaining chalcones as possible anti-inflammatory and antioxidant agents. Bioorg Med Chem Lett.

[11] Vogel S, Barbic M, Jürgenliemk G, Heilmann J (2010). Synthesis, cytotoxicity, anti-oxidative and anti-inflammatory activity of chalcones and influence of A-ring modifications on the pharmacological effect. Eur J Med Chem.

[12] Tran T-D, Park H, Kim HP, Ecker GF, Thai K-M (2009). Inhibitory activity of prostaglandin E2 production by the synthetic 21-hydroxychalcone analogues: synthesis and SAR study. Bioorg Med Chem Lett.

[13] Kim BT, Kwang-Joong O, Chun JC, Hwang KJ (2008). Synthesis of dihydroxylated chalcone derivatives with diverse substitution patterns and their radical scavenging ability toward DPPH free radicals. Bull Korean Chem Soc.

[14] Doan TN, Tran T-D (2011). Synthesis, antioxidant and antimicrobial activities of a novel series of chalcones, pyrazolic chalcones, and allylic chalcones. Pharmacol Pharm.

[15] Sivakumar PM, Prabhakar PK, Doble M (2011). Synthesis, antioxidant evaluation, and quantitative structure–activity relationship studies of chalcones. Med Chem Res.

[16] Vogel S, Ohmayer S, Brunner G, Heilmann J (2008). Natural and non-natural prenylated chalcones: synthesis, cytotoxicity and anti-oxidative activity. Bioorg Med Chem.

[17] Echeverria C, Santibañez JF, Donoso-Tauda O, Escobar CA, Ramirez-Tagle R (2009). Structural antitumoral activity relationships of synthetic chalcones. Int J Mol Sci.

[18] Modzelewska A, Pettit C, Achanta G, Davidson NE, Huang P, Khan SR (2006). Anticancer activities of novel chalcone and bis-chalcone derivatives. Bioorg Med Chem.

[19] Vogel S, Heilmann J (2008). Synthesis, cytotoxicity, and antioxidative activity of minor prenylated chalcones from *Humulus lupulus*. J Nat Prod.

[20] Kamal A, Kashi Reddy M, Viswanath A (2013). The design and development of imidazothiazole-chalcone derivatives as potential anticancer drugs. Expert Opin Drug Discov.

[21] Do TH, Nguyen DM, Truong VD, Do THT, Le MT, Pham TQ, Thai KM, Tran TD (2016). Synthesis and selective cytotoxic activities on rhabdomyosarcoma and noncancerous cells of some heterocyclic chalcones. Molecule.

[22] Neves MP, Lima RT, Choosang K, Pakkong P, de São José Nascimento M, Vasconcelos MH, Pinto M, Silva AM, Cidade H (2012). Synthesis of a natural chalcone and its prenyl analogs—evaluation of tumor cell growth-inhibitory activities, and effects on cell cycle and apoptosis. Chem Biodivers.

[23] Forejtníková H, Lunerová K, Kubínová R, Jankovská D, Marek R, Suchý V, Vondrácek J (2005). Chemoprotective and toxic potentials of synthetic and natural chalcones and dihydrochalcones in vitro. Toxicology.

[24] Orlikova B, Tasdemir D, Golais F, Dicato M, Diederich M (2011). Dietary chalcones with chemopreventive and chemotherapeutic potential. Genes Nutr.

[25] Ekhlass N, El-Badry YA, Eltoukhy AMM, Ayyad RR (2016). Synthesis and antiproliferative activity of 1-(4-(1H-Indol-3-Yl)-6-(4-Methoxyphenyl)Pyrimidin-2-yl)hydrazine and its pyrazolo pyrimidine derivatives. Medicinal chemistry. Med chem.

[26] Murakami N, Takase H, Saito T, Iwata K, Miura H, Naruse T (1998). Effects of a novel non-steroidal anti-inflammatordrug (M-5011) on bone metabolism in rats with collageninduced arthritis. Eur J Pharmacol.

[27] Kulandasamy R, Adhikari AV, Stables JP (2009). A new class of anticonvulsants possessing 6Hz activity: 3,4-dialkyloxy thiophene bishydrazones. Eur J Med Chem.

[28] Lu X, Wan B, Franzblau SG, You Q (2011). Design, synthesis and anti-tubercular evaluation of new 2-acylated and 2-alkylated amino-5-(4-(benzyloxy) phenyl)thiophene-3-carboxylic acid derivatives. Part 1. Eur J Med Chem.

[29] Kaushik NK, Kim HS, Chae YJ (2012). Synthesis and anticancer activity of di(3-thienyl)methanol and di(3-thienyl)methane. Molecules.

[30] Shehab WS, Saad HA, Mouneir SM (2017). Synthesis and antitumor/antiviral evaluation of 6-thienyl-5-cyano-2-thiouracil derivatives and their thiogalactosides analogs. Curr Org Synth.

[31] Shehab WS, Mouneir SM (2015). Design, synthesis, antimicrobial activity and anticancer screening of some new1,3-thiazolidin-4-ones derivatives. Eur J Chem.

[32] Shehab WS (2009). Synthesis of tetrahydropyrimidine derivatives and its glycosides. Curr Org Chem.

[33] Corral C, Lissavetzky J, Manzanares I (1999). Synthesis and preliminary pharmacological evaluation of thiophene analogues of viloxazine as potentialantidepressant drugs. Bioorg Med Chem.

[34] Abdel-Wahab BF, Abdel-Aziz HA, Ahmed EM (2009). Synthesis and antimicrobial evaluation of 1-(benzofuran-2-yl)-4-nitro-3-arylbutan-1-ones and 3-(benzofuran-2-yl)-4, 5-dihydro-5-aryl-1-[4-(aryl)-1, 3-thiazol-2-yl]-1H-pyrazoles. Eur J Med Chem.

[35] Kulmacz RJ, Lands WEM (1983). Requirements for hydroperoxide by the cyclooxygenase and peroxidase activities of prostaglandin H synthase. Prostaglandins.

[36] Jacob J, Prakash Kumar B (2015). Dual COX/LOX inhibition: screening and evaluation of effect of medicinal plants of Kerala as Antiinflammatory agents Pharmacogn. Phytochemistry.

[37] Kuroda T, Suzuki F, Tamura T, Ohmori K, Hosoe H (1992). A novel synthesis and potent antiinflammatory activity of 4-hydroxy-2(1H)-oxo-1-phenyl-1,8-naphthyridine-3-carboxamides. J Med Chem.

[38] Simone RD, Chini MG, Bruno I, Riccio R (2011). Benzimidazole-1,2,3-triazole hybrid molecules: synthesis and evaluation for antibacterial/antifungal activity. J Med Chem.

[39] Khalil NA, Ahmed EM, Mohamed KO, Nissan YM (2014). Synthesis and biological evaluation of new pyrazolone–pyridazine conjugates as anti-inflammatory and analgesic agents. Bioorg Med Chem.

[40] Lokwani D, Azad R, Sarkate A, Reddanna P, Shinde D (2015). Structure based library design (SBLD) for new 1, 4-dihydropyrimidine scaffold as simultaneous COX-1/COX-2 and 5-LOX inhibitors. Bioorg Med Chem.

[41] Abdelgawad MA, Bakr RB, Azouz AA (2018). Novel pyrimidine-pyridine hybrids: synthesis, cyclooxygenase inhibition, anti-inflammatory activity and ulcerogenic liability. Bioorg Chem.

[42] Nam TG, Nara SJ, Zagol-Ikapitte I, Cooper T, Valgimigli L, Oates JA, Porter NA, Boutaud O, Pratt DA (2009). Pyridine and pyrimidine analogs of acetaminophen as inhibitors of lipid peroxidation and cyclooxygenase and lipoxygenase catalysis. Org Biomol Chem.

[43] Kotb ER, Soliman HA, Morsy EMH, Abdelwahed NAM (2017). New pyridine and triazolopyridine derivatives: synthesis, antimicrobialand antioxidant evaluation. Acta Pol Pharm.

[44] Mojarrab M, Soltani R, Aliabadi A (2013). Pyridine based chalcones: synthesis and evaluation of antioxidant activity of 1-phenyl-3-(pyridin-2-yl)prop-2-en-1-one derivatives. Jundishapur J Nat Pharm Prod.

[45] Rani J, Saini M, Kumar S, Verma PK (2017). Design, synthesis and biological potentials of novel tetrahydroimidazo[1,2-a]pyrimidinederivatives. Chem Cent J.

